# Recentering on the Essentials: A Multicenter Cross‐Sectional Survey of Academic Coordinators’ Perspectives on Student Self‐Care in Nursing Curricula

**DOI:** 10.1155/nrp/8200644

**Published:** 2026-10-06

**Authors:** Made Rini Damayanti, Kaori Shimoinaba, Katrina Recoche

**Affiliations:** ^1^ School of Nursing and Midwifery Faculty of Medicine Nursing and Health Sciences Monash University Clayton Australia monash.ac.za; ^2^ Department of Nursing Faculty of Medicine Universitas Udayana Bali Indonesia

**Keywords:** curriculum, holistic, integral nursing, nursing education, self-care, undergraduate nursing student

## Abstract

**Introduction:**

The increasing complexities and demands of modern nursing practice necessitate a shift in educational priorities toward a more holistic and integrated model. This approach promotes the concurrent development of students’ theoretical knowledge, clinical skills, and self‐nurturing abilities. As key stakeholders, academic coordinators play a central role in incorporating nursing student self‐care into education; however, their perspectives have been underrepresented in the existing research.

**Aim:**

Informed by Dossey’s theory of integral nursing, this study aimed to explore academic coordinators’ perspectives on how nursing student self‐care has been taught and assessed in participating Indonesian nursing education institutions.

**Methods:**

A multicenter cross‐sectional descriptive study incorporating quantitative and qualitative approaches was conducted. An online survey with both closed and open‐ended questions was used to collect data across 13 institutions, with a total 25 academic coordinators invited to participate. Data were analyzed through descriptive statistics and summative content analysis. Responses were analyzed at the academic coordinator level.

**Results:**

The intention–action gap, which illustrates the divergence between what respondents rated as necessary to teach and their actual self‐care teaching practice yielded positive values across all self‐care dimensions. Three key themes were identified: navigating challenges, differences in teaching and assessment approaches, and envisioning future change.

**Conclusion:**

The findings suggest that less emphasis was placed on teaching nursing student self‐care across the participating institutions, with opportunities for improvement across all dimensions. Discrepancies between the two datasets also emerged, potentially reflecting a complex interplay between structural and cultural dimensions shaping self‐care. Nursing student self‐care integration into education should go beyond isolated initiatives to foster synergetic structural and cultural transformations to create a supportive learning environment that embodies and reinforces self‐care. This will encourage students to recognize self‐care as a foundation for personal well‐being and professional practice, aligning with contemporary nursing education’s core value of caring for others and oneself.

## 1. Introduction

Nurse self‐care refers to nurses purposefully valuing themselves and consistently engaging in behaviors that support their health, well‐being, coping strategies, and resilience [1, 2]. With nurses forming the backbone of healthcare systems globally, accounting for nearly 60% health workforce [3], the sustainability of healthcare provision depends on their well‐being and performance, making nurse self‐care remains a relevant topic in current and future discourse. The International Council of Nurses (ICN) advances nurse self‐care by reinforcing the need to teach nursing students its concept and foster the acquisition of self‐nurturing behaviors by nurses during their education program [4]. Regulatory bodies in every country need to explicitly guide nursing education institutions on incorporating nurse self‐care components into their curricula [5]. Unfortunately, nursing standards in individual countries do not necessarily align with those of the ICN. Throughout this study, the term nursing student self‐care is used to describe the self‐care practices of nursing students.

## 2. Background and Literature Review

While managing the intense demands of academic and clinical placements and meeting professional standards and expectations, nursing students’ health and well‐being may be given lower priority [6, 7]. If left unaddressed, students may develop psychological distress manifesting as a range of emotional, cognitive, behavioral, and physical symptoms; which poses a significant threat not only to the individuals themselves but also to educational institutions, patient outcomes, healthcare organizations, and the long‐term sustainability of the healthcare systems [8, 9]. Growing awareness of the value of nursing student self‐care has led to a greater focus on transforming nursing education to better support students in becoming competent nurses who thrive both professionally and personally [10, 11]. This shift requires reorientating nursing education around its values of caring and holistic practice, by recognizing that nurses’ health and well‐being are integral to the care they provide, and self‐care should be fostered from the outset of professional education [4, 12].

A holistic nursing approach to self‐care is especially relevant to current nursing education and practice, which seek to educate the “whole” nurse [2]. This holistic perspective on nursing student self‐care is further reinforced by established holistic nursing theories that provide a robust conceptual foundation for integrating the concept into nursing programs. Watson’s theory of human caring, for example, emphasizes the reciprocal relationship between a nurse’s personal well‐being and their professional performance [13]. Dossey’s theory of integral nursing (TIN) offers a contemporary framework by viewing nurse self‐care as a healing practice that aims to restore nurses’ wholeness, encompassing all aspects of the “self.” It promotes a balanced state and integrated functioning of the body, mind, and spiritual and social selves of nurses, essential for facilitating the healing of others [1]. Drawing from Ken Wilber’s integral theory, TIN maps the complexity of human experiences into four interrelated quadrants of realities [14, 15], ensuring a holistic and balanced perspective (see Figure 1). Nurse self‐care is regarded as a multidimensional practice that extends beyond individual responsibility, involving relational, organizational, and systemic factors that demand collective responsibility and supportive healing environments [14].

**FIGURE 1:**
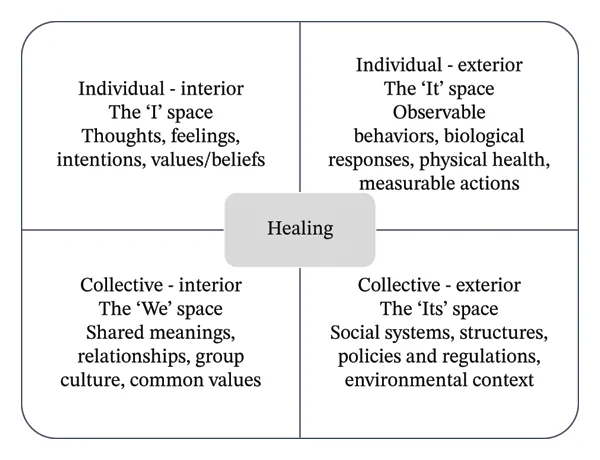
Dossey’s four quadrants of reality in theory of integral nursing. *Note.* Adapted from Dossey’s Theory of Integral Nursing. Source: Dossey, B.M. (2020). Theory of integral nursing. In M.B. Helming, D.A. Shields, K.M. Avino, & W.E. Rosa (Eds.), Dossey & Keegan’s holistic nursing: A handbook for practice (8^th^ ed., pp. 53–75). Jones & Bartlett Learning.

Dossey’s TIN has the potential to provide a rational and structured framework for designing educational experiences that develop students’ ability to deliver compassionate nursing care while cultivating their own well‐being and resilience [1]. However, empirical research on integrating nurse self‐care into nursing education through the lens of TIN remains scarce. One documented example is the Northern New Mexico College’s Registered Nurse‐to‐Bachelor Science Nursing Program, which explicitly embeds holistic self‐care throughout its curriculum by aligning all courses with TIN principles [16, 17]. The existing literature, while not specifically informed by TIN, also suggests that research on nursing student self‐care has predominantly been explored from the perspectives of students and frontline educators [18, 19]. This indicates a gap in understanding the administrative, structural, cultural, and pedagogical conditions surrounding nursing student self‐care within the curricula from the perspective of key academic stakeholders. A literature search on this topic identified one Canadian‐based study [5], which is among the few empirical works examining how nursing student self‐care competencies are taught and assessed within education institutions. While their insights offer meaningful contributions, only eight program directors participated, limiting the range of perspectives to understand practical aspects of nursing self‐care. Accordingly, evidence from diverse educational contexts, including Indonesia, is needed to determine how nursing student self‐care is understood and embedded within different educational, policy, and broader sociocultural environments.

A review of the Indonesian Nursing Professional Standards and Code of Ethics found no explicit reference to nurse self‐care competencies (Ministry of Health of the Republic of Indonesia (MoHRI), 2020). Similarly, the Indonesian entry‐to‐practice professional nursing degree core curriculum emphasizes clinical competence, professionalism, patient‐centered care, and ethics, without explicitly identifying nursing student self‐care as a curriculum competency [20]. While these national documents suggest limited explicit attention to nurse self‐care, Indonesian nursing education institutions have autonomy to supplement the national curriculum with additional content aligned with institutional needs and priorities [21]. This institutional autonomy, coupled with the rapid expansion of nursing education in Indonesia, with more than 850 nursing institutions [22, 23], may lead to diversity in how nursing student self‐care is addressed across institutions [24]. In light of studies reporting a substantial burden of psychological distress and burnout among Indonesian nursing students and nurses [25, 26], these contextual factors underscore the need to examine self‐care within Indonesian nursing education.

Although the importance of nursing student self‐care is increasingly recognized, there is limited understanding of how it is enacted within the nursing curriculum from the perspective of academic leaders responsible for curriculum design and implementation, particularly in Indonesia. Research to date has concentrated on individual behaviors rather than the educational culture and organizational systems that underpin nursing student self‐care. The TIN helps address this gap by providing a framework for understanding the collective interior, or the “We” space (shared educational values, meanings, and culture) and collective exterior, or the “Its” space (policies, curriculum structures, organizational system, and institutional practices) dimensions of nursing education [27]. Mapping and exploring the phenomenon within the TIN collective quadrants supported a comprehensive and multidimensional understanding, by placing nursing student self‐care within its broader contextual determinants; thus, preserving the theory’s holistic principles. The selective application of the theory did not undermine its structure, but instead utilized it strategically to explore the most relevant aspects for the study [28]. Using the two collective quadrants, this study sought to clarify how nursing student self‐care is viewed, taught, and assessed, from the perspectives of academic coordinators, whose roles reflect and are inherently embedded within the collective context.

## 3. Methods

### 3.1. Design

The study employed a descriptive cross‐sectional study design. This article was prepared following the Consensus‐Based Checklist for Reporting of Survey Studies (CROSS) guidelines [29].

### 3.2. Respondents and Setting

This study was conducted at tertiary education institutions offering entry‐to‐practice professional degrees (Bachelor of Nursing/BSN leading to Professional Nurses/PN) that are members of the Association of Indonesian Nurse Education Center (AINEC) Regional X. The BSN leading to PN program is a 5‐year nursing education program consisting of a 4‐year academic phase (BSN) and a mandatory 1‐year clinical and community‐focused placement phase (PN) [20]. As the regional extension of AINEC Headquarters, AINEC Regional X is instrumental in promoting quality nursing education across the region by providing curriculum frameworks and technical support to its member institutions. The AINEC Regional X network includes 21 institutions scattered across Bali, West Nusa Tenggara, and East Nusa Tenggara Provinces (AINEC, 2023), representing an estimated population base of 15.74 million individuals [30].

Institutions were selected using a purposive sampling approach based on the criteria of offering both BSN and PN programs and having students enrolled in all year levels. Therefore, newly established institutions were excluded because they are not yet fully operational. Fifteen out of 21 AINEC Regional X member institutions at the time of the study met the eligibility criteria. Two institutions declined to participate, leaving 13 institutions included, with a total of 6739 students. Academic coordinators overseeing both BSN and PN programs in the 13 institutions were recruited using a total population sampling method. Academic coordinators serve as primary stakeholders in Indonesian nursing education institutions who are well positioned to provide valuable insights into current practices and the factors that facilitate or hinder nursing student self‐care integration. While specific roles may vary across settings, academic coordinators are typically tasked with overseeing curriculum and assessment; managing academic administrative and operational functions; supporting both academic staff/educators and students; and maintaining academic integrity [31].

### 3.3. Data Collection and Ethical Considerations

This study used a web‐based survey, Qualtrics Plus (Qualtrics, Provo, UT), and employed a tool modified from a Canadian study [5]. The instrument, which included a mix of closed‐ and open‐ended questions, covered nine nurse self‐care dimensions, as shown in Table 1. The overall definitions of self‐care and its nine dimensions in the survey instrument align well with the holistic self‐care definition and the theoretical framework underpinning the current study. The combination of closed‐ and open‐ended questions enabled the instrument to simultaneously generate complementary data and facilitated the exploration of the TIN’s collective quadrants [1]. The 13 closed‐ended questions asked about the type of nursing program (BSN, PN), nursing student self‐care competencies (nine dimensions, as displayed in Table 2), information on how nursing student self‐care competencies were taught and assessed (timing, pedagogical strategies, instructional methods, and designated personnel), and respondents’ attitudes about them (levels of satisfaction, perceived difficulty, and perceived significance). Finally, the last two questions focused on exploring challenges and facilitators identified by academic coordinators regarding experiences in teaching and assessing nursing student self‐care to students and their general views about it [5].

**TABLE 1:** Characteristics of respondent (*n* = 25).

Characteristics	Frequency (*n*)	%
Current position title		
Bachelor of Science in Nursing (BSN) Program Academic Coordinator	12	48
Professional Nurse (PN) “Ners” Program Academic Coordinator	12	48
BSN‐PN Program Academic Coordinator	1	4
Institution location		
Bali Province	6	46.15
West Nusa Tenggara Province	4	30.77
East Nusa Tenggara Province	3	23.08

**TABLE 2:** Nursing student self‐care teaching and assessment (*n* = 25).

	Nursing student self‐care dimensions								
	Assessment of personal health & well‐being	Resilience to cope with stressful situations	Capacity to navigate self‐care resources	Professional self‐care	Physical self‐care	Psychological self‐care	Emotional self‐care	Spiritual self‐care	Relationship self‐care
Teaching frequency									
Yes	18 (72%)	19 (76%)	13 (52%)	19 (76%)	19 (76%)	18 (72%)	16 (64%)	17 (68%)	15 (60%)
No	7 (28%)	6 (24%)	12 (48%)	6 (24%)	6 (24%)	7 (28%)	9 (36%)	8 (32%)	10 (40%)
Importance of nursing student self‐care in the curriculum									
Not at all	—	—	—	—	—	—	—	—	—
Low	—	—	—	—	—	—	—	—	—
Slightly	—	—	—	—	—	—	—	—	—
Moderately	6 (24%)	2 (8%)	5 (20%)	4 (16%)	4 (16%)	2 (8%)	2 (8%)	1 (4%)	4 (16%)
Very	13 (52%)	17 (68%)	15 (60%)	11 (44%)	13 (52%)	13 (52%)	14 (56%)	14 (56%)	14 (56%)
Extremely	6 (24%)	6 (24%)	5 (20%)	10 (40%)	8 (32%)	10 (40%)	9 (36%)	10 (40%)	7 (28%)
Period of teaching^∗^									
Semester 1	15	9	12	10	12	7	8	11	9
Semester 2	8	5	3	6	6	4	4	6	6
Semester 3	3	3	3	2	3	5	4	4	3
Semester 4	4	7	4	3	3	7	5	5	5
Semester 5	4	4	2	3	2	3	3	4	2
Semester 6	2	1	1	2	2	2	2	4	3
Semester 7	2	1	1	1	1	1	1	3	3
Semester 8	3	2	4	3)	3	3	4	6	3
Professional Nursing	5	3	3	5	4	4	4	4	5
Strategy of teaching^∗^									
Formally in class	14	10	10	15	14	15	14	15	13
Formally in clinical setting	7	9	6	10	9	9	8	7	9
Informally in class	9	12	9	9	11	12	10	10	11
Informally in clinical setting	6	10	8	6	8	9	7	6	7
Method of teaching^∗^									
Case study/critical incident	9	10	10	11	12	11	9	9	7
Group discussion	13	12	11	12	12	12	10	11	9
Lecture	12	10	11	10	13	14	12	16	10
Reflection	11	11	9	7	9	11	9	10	10
Simulation/roleplay	6	5	6	6	6	8	8	6	8
Student–educator interaction	11	13	11	11	11	11	11	10	12
Tutorial/seminar	2	3	2	3	3	3	3	3	3
Person to teach^∗^									
Faculty within nursing program	20	21	17	21	21	20	20	19	19
Faculty outside nursing program	3	4	3	2	2	6	6	8	3
Nurses in the clinical setting	7	9	6	8	9	9	7	7	7
Other healthcare staff	2	2	2	1	2	2	1	1	1
Teaching satisfaction									
Very dissatisfied	1 (4%)	1 (4%)	1 (4%)	1 (4%)	1 (4%)	1 (4%)	1 (4%)	1 (4%)	1 (4%)
Dissatisfied	3 (12%)	1 (4%)	4 (16%)	3 (12%)	1 (4%)	3 (12%)	2 (8%)	2 (8%)	2 (8%)
Satisfied	10 (40%)	11 (44%)	8 (32%)	10 (40%)	12 (48%)	12 (48%)	12 (48%)	10 (40%)	9 (36%)
Very satisfied	5 (20%)	6 (24%)	5 (20%)	5 (20%)	6 (24%)	5 (20%)	4 (16%)	7 (28%)	5 (20%)
N/A	6 (24%)	6 (24%)	7 (28%)	6 (24%)	5 (20%)	4 (16%)	6 (24%)	5 (20%)	3 (12%)
Teaching difficulty									
Very easy	—	—	—	—	—	—	—	—	—
Easy	14 (56%)	12 (48%)	13 (52%)	14 (56%)	21 (84%)	11 (44%)	10 (40%)	13 (52%)	13 (52%)
Difficult	11 (44%)	13 (52%)	12 (48%)	11 (44%)	4 (16%)	12 (48%)	14 (56%)	12 (48%)	10 (40%)
Very difficult	—	—	—	—	—	2 (8%)	1 (4%)	—	2 (8%)
Method of assessment^∗^									
Patient feedback	6	6	5	6	6	7	7	7	7
Educator feedback	16	16	15	13	16	17	16	16	14
Peer feedback	6	6	7	6	8	8	7	6	9
Course assignments	8	8	9	10	10	10	8	8	7
Quizzes/tests	8	8	8	10	10	10	10	8	9
Reflective journal	1	1	1	3	3	3	4	3	4
Portfolios	2	2	2	2	2	2	2	2	2
Person to assess^∗^									
Faculty within nursing program	20	18	17	20	20	19	18	16	18
Faculty outside nursing program	3	5	4	3	3	5	4	5	4
Nurses in the clinical setting	8	6	5	8	9	8	7	7	8
Other healthcare staff	1	1	1	1	1	1	1	1	1
Assessment satisfaction									
Very dissatisfied	2 (8%)	1 (4%)	2 (8%)	2 (8%)	2 (8%)	1 (4%)	1 (4%)	1 (4%)	1 (4%)
Dissatisfied	4 (16%)	2 (8%)	3 (12%)	3 (12%)	2 (8%)	3 (12%)	3 (12%)	2 (8%)	3 (12%)
Satisfied	8 (32%)	10 (40%)	9 (36%)	11 (44%)	12 (48%)	12 (48%)	10 (40%)	12 (48%)	8 (32%)
Very satisfied	4 (16%)	4 (16%)	2 (8%)	2 (8%)	4 (16%)	4 (16%)	3 (12%)	4 (16%)	4 (16%)
N/A	7 (28%)	8 (32%)	9 (36%)	7 (28%)	5 (20%)	5 (20%)	8 (32%)	6 (24%)	9 (36%)
Assessment difficulty									
Very easy	1 (4%)	—	1 (4%)	2 (8%)	2 (8%)	—	—	—	1 (4%)
Easy	14 (56%)	11 (44%)	12 (48%)	12 (48%)	16 (64%)	11 (44%)	11 (44%)	11 (44%)	10 (40%)
Difficult	10 (40%)	12 (48%)	11 (44%)	11 (44%)	7 (28%)	13 (52%)	13 (52%)	12 (48%)	12 (48%)
Very difficult	—	2 (8%)	1 (4%)	—	—	1 (4%)	1 (4%)	2 (8%)	2 (8%)

^∗^Allowed multiple responses.

Following permission from the instrument’s developers and before using it in the Indonesian population, the instrument underwent cross‐cultural adaptation (CCA), following steps recommended by Beaton et al. [32]. The process involved rigorous forward and backward translations, expert committee reviews, pilot testing of the pre‐final Bahasa version, and coordination with the instrument developers. All of these steps yielded satisfactory results, as indicated by item‐level content validity index (I‐CVI) values of 0.857 for 1 item and 1.00 for the other 14 items, and a scale‐level CVI score of 0.994. Ten nurse educators were invited to pilot the instrument, which yielded an inter‐rater agreement of 94.67%. Pilot testing feedback prompted revisions to several items. The revision involved refining the description of nursing student self‐care and its nine dimensions to place greater emphasis on student self‐care, which was presented on the first page of the questionnaire. Once reevaluated by the reviewers, the final version was used for the actual data collection.

The study received ethics approval from the Human Research Ethics Committees of Monash University (approval number: 40,946) and Universitas Udayana (approval number: 2602/UN14.2.2.VII.14/LT/2023). Indonesian local authorities and participating institutions granted research permits. Research liaisons at participating institutions facilitated the distribution of the survey link to academic coordinators. An ad hoc online information session was conducted to ensure respondents understood the purpose of the study, namely, to explore student self‐care in nursing education. This emphasis on student self‐care was further reiterated in the explanatory statement, informed consent, survey invitation message, and the main questionnaire. Informed consent was obtained from academic coordinators who were aware of their voluntary and anonymous participation. Given the ethical considerations and the potential risk of re‐identification, no personal information was obtained. Data collection took place between January and April, 2024.

### 3.4. Data Analysis

Responses were analyzed at the individual academic coordinator level; therefore, the findings reflect their individual perceptions rather than institutional‐level characteristics or practices. Data generated from the closed‐ended questions were analyzed descriptively for frequencies and percentage distributions using the IBM Statistical Package for Social Sciences (SPSS) Statistics program, version 29 for Windows. In addition, an intention–action gap was calculated by subtracting the relative teaching frequencies from the relative importance frequencies (yielded from converting the weighted mean). The gap illustrated the divergence between what the respondents rated as necessary (intention) versus their actual self‐care teaching practice (action) [5, 33]. Weighted mean scores were calculated by following the scoring procedure of the original instrument [5]. Responses on the six‐point Likert scale were assigned numerical values from 1 (“not at all important”) to 6 (“extremely important”). The weighted mean was calculated by multiplying the frequency of responses in each category by its corresponding numerical value, summing these products, and dividing by the total number of responses. The weighted mean was then converted to a relative importance frequency by dividing it by the maximum possible score (6) and multiplying the result by 100 to express it as a percentage. The relative teaching frequency was calculated by dividing the number of respondents selecting each teaching frequency (option yes) for the corresponding dimension by the total number of respondents and multiplying the result by 100 [5]. The formulas and calculations for the weighted mean, relative importance, relative teaching, and intention–action gap are provided in Table 3.

**TABLE 3:** Intention–action gap in nursing student self‐care (*n* = 25).

	Likert numerical value (Xi)	Assessment of personal health and well‐being (*f* *i*)	Resilience to cope with stressful situations	Capacity to navigate self‐care resources	Professional self‐care	Physical self‐care	Psychological self‐care	Emotional self‐care	Spiritual self‐care	Relationship self‐care
The importance of nursing self‐care										
Not at all important	1	—	—	—	—	—	—	—	—	—
Less important	2	—	—	—	—	—	—	—	—	—
Slightly important	3	—	—	—	—	—	—	—	—	—
Moderately important	4	6	2	5	4	4	2	2	1	4
Very important	5	13	17	15	11	13	13	14	14	14
Extremely important	6	6	6	5	10	8	10	9	10	7
Weighted mean^a^: $\overset{¯}{X}w=\left(\Sigma \left(fi\times Xi\right)\right)/\left(\Sigma fi\right)$		5	5.16	5	5.24	5.16	5.32	5.28	5.36	5.12
Relative importance^b^ (%) RI $=\left(\left(\overset{¯}{X}w\right)/6\right)\times 100$		83.33	86.00	83.33	87.33	86.00	88.87	88.00	89.93	85.33

Teaching frequency		18	19	13	19	19	18	16	17	15
Relative teaching^c^ (%) $\text{RT}=\left(\left(\overset{¯}{X}w\right)/6\right)\times 100$		72	76	52	76	76	72	64	68	60

Intention–action gap^d^ (%) IAG = RI − RT		11.33	10.00	31.33	11.33	10.00	16.67	24.00	21.93	25.33

*Note:* $\overset{¯}{X}w$ is the weighted mean; *f* *i* is the frequency of responses in category I; Xi is the numerical score assigned to that category; RI is relative importance; RT is relative teaching; and IAG is the intention–action gap. Intention–action gap (per each self‐care dimension) is calculated by subtracting the percentage reporting teaching (action) from the percentage rating importance of self‐care in the nursing curriculum (intention). A positive gap indicates a higher rated importance than teaching dedicated to self‐care dimension, implying underemphasis in teaching self‐care.

^a^Weighted mean.

^b^Relative importance.

^c^Relative teaching.

^d^Intention–action gap.

The relatively brief nature of the open‐ended responses informed the selection of summative content analysis as an appropriate approach for systematically identifying and interpreting of recurring concepts and patterns across the dataset [34]. Two professional translators were involved in translating the data from Bahasa into English; one translator verified the accuracy of the other’s translation results. Employing summative content analysis, the researchers could count and compare the frequency of particular words or contents and their alternative terms appearing in the data across coding categories and finally explore their usage. This was followed up by interpreting the context or underlying meanings of these words or contents to understand the situation under study [34]. In this study, the unit of analysis was the meaning unit, defined as a phrase or sentence expressing a discrete idea or concept within individual open‐ended survey responses. Within each response, meaningful concepts were identified and assigned one or more code labels where applicable. Codes and categories were identified both deductively, informed by the study’s aims and theoretical framework, and inductively, based on insights emerging from respondents’ responses [35]. All three of the research team members conducted the analysis independently by first carefully reading and re‐reading the whole dataset to gain a comprehensive understanding. A respondent was only coded once for the same topic even if they mentioned the same topic multiple times [36]. The coded responses were compared for validation purposes through iterative and collaborative reviews, and any discrepancies were discussed until consensus was reached. Once identified, the similar codes were sorted into broader conceptual categories or subcategories representing related concepts and patterns within the dataset, which were quantified and organized into themes [34]. Categories and subcategories were developed collaboratively through regular team discussions, and any differences in interpretation were resolved through consensus. The findings were illustrated by relevant quotations taken from the transcripts. The summative content analysis was conducted iteratively [34], and reflective journaling was conducted throughout the process by the primary researcher. All stages of the analysis were undertaken manually using Microsoft Word for coding and data organization.

## 4. Results

### 4.1. Closed‐Ended Survey

All academic coordinators for both BSN and PN programs (*n* = 25) across 13 invited institutions completed the survey, giving a 100% response rate. A high response rate to surveys is commonplace in Indonesian nursing research [37]. In contrast to the 12 institutions where a different person coordinated each BSN or PN program, one participating institution in this study had one individual responsible for academic oversight of both programs (see Table 1).

Although the study included 13 institutions, the findings presented in Table 2 reflects perspectives of 25 BSN and PN academic coordinators on how nursing student self‐care was taught and assessed in the respective institutions. Resilience to cope with stressful situations and professional and physical self‐care were the most frequently taught nursing student self‐care dimensions. By comparison, the capacity to navigate self‐care resources emerged as the least frequently taught dimension. All academic coordinators who reported teaching self‐care competencies to students indicated that teaching largely occurred during the first semester of nursing education for all nine dimensions. Respondents reported using formal and informal methods to teach nursing student self‐care in class and clinical settings. Though most teaching occurred formally in class, the most utilized teaching strategies were group discussion, lectures, and student–educator interaction. Meanwhile, the least utilized methods were tutorial/seminar and simulation/role‐play. Feedback from the educator was named as the most frequently used assessment method across all nursing student self‐care dimensions. Contrary to this, reflective journals and portfolios were ranked as the least commonly used assessment method. In all dimensions, faculty within the nursing program was primarily responsible for teaching and assessing nursing student self‐care competencies.

All nursing student self‐care dimensions in this study yielded positive intention–action gap values (see Table 3), indicating that potentially less emphasis was placed on teaching all self‐care dimensions to students across the participating institutions. This study highlights what teaching areas needed to improve the most: the capacity to navigate self‐care resources and the dimensions of relationships and emotional self‐care. By contrast, resilience to cope with stressful situations and physical self‐care were perceived as the least urgent teaching areas to enhance. Table 3 shows inconsistent results between nursing student self‐care dimensions rated as the most often taught, such as resilience to cope with stressful situations and professional and physical self‐care, and those considered necessary within the Indonesian nursing curriculum by academic coordinators. The latter is reflected in the weighted mean importance of nursing student self‐care, with spiritual, psychological, and emotional self‐care identified as the top three dimensions based on their significance in nursing education. The dimension with the lowest teaching frequency, the capacity to navigate self‐care resources, was regarded as the least essential to include in the curriculum. Interestingly, the assessment of personal health and well‐being was deemed the least important dimension to teach, yet this was one of the most frequently taught dimensions.

The academic coordinators’ satisfaction and perceptions of the difficulty of teaching and assessing nursing student self‐care competencies are displayed in Table 4. The respondents reported a higher satisfaction score with how nursing student self‐care was taught to students than with how it was assessed across nine dimensions. Teaching nursing student self‐care in physical, psychological, and spiritual dimensions was rated as the most satisfying teaching area, while the highest‐rated dimension for assessment was psychological self‐care. The most unsatisfying teaching and assessing areas were relationship and capacity to navigate self‐care resources. Table 4 also demonstrates that assessing nursing student self‐care was graded as more challenging than teaching it. While psychological and emotional self‐care dimensions were considered the most difficult areas to teach, resilience to cope with stressful situations and spirituality were claimed as the most complex dimensions to assess. Physical self‐care was perceived as the most effortless dimension in both practice areas.

**TABLE 4:** Satisfaction and difficulty in nursing student self‐care teaching and assessment (*n* = 25).

Nursing student self‐care dimensions	Teaching satisfaction (weighted mean)	Assessment satisfaction (weighted mean)	Teaching difficulty (weighted mean)	Assessment difficulty (weighted mean)
Assessment of personal health and well‐being	2.28	2.00	2.44	2.36
Resilience to cope with stressful situations	2.40	2.04	2.52	2.64
Capacity to navigate self‐care resources	2.12	1.72	2.48	2.48
Professional self‐care	2.28	1.96	2.44	2.36
Physical self‐care	2.52	2.32	2.16	2.20
Psychological self‐care	2.52	2.36	2.64	2.60
Emotional self‐care	2.28	1.96	2.64	2.60
Spiritual self‐care	2.52	2.28	2.48	2.64
Relationship self‐care	2.08	1.88	2.56	2.60
Total	2.33	2.06	2.48	2.50

*Note:* Numerical values for the five‐point Likert scale of satisfaction with teaching and assessment of nursing student self‐care are as follows: 1 for “very dissatisfied,” 2 for “dissatisfied,” 3 for “satisfied,” 4 for “very satisfied,” and 0 for N/A. For teaching and assessment difficulty, the numerical values were assigned as follows: 1 (“very easy”), 2 (“easy”), 3 (“difficult”), and 4 (“very difficult”). The weighted mean was calculated using the same formula as for self‐care importance (see Table 3).

### 4.2. Open‐Ended Survey

Twenty two out of 25 respondents provided responses to the open‐ended questions. Participants 6, 7, and 10 did not provide comments in this section. Summative content analysis identified three key themes: (1) navigating challenges; (2) differences in teaching and assessment approaches; and (3) envisioning future change. Table 5 displays the identified themes, categories, and subcategories, along with their total frequency and percentage of appearance in the dataset.

**TABLE 5:** Summative content analysis results (*n* = 22).

Themes	Categories‐subcategories	*n* = 22	%
Navigating challenge	*Students-related challenges*	8	36.36
	Diverse sociodemographic characteristics	5	22.73
	Diverse characters/personalities	3	13.64
	*Educators-related challenges*	5	22.73
	Diverse skills and knowledge to teach self‐care	5	22.73
	*Organizational-related challenges*	10	45.45
	Inadequate guidelines	6	27.27
	Excessive/extra workload	4	18.18
	*No challenges*	4	18.18

Differences in teaching and assessment approaches	No teaching and assessment	4	18.18
	Informally taught and assessed	5	22.73
	Taught and assessed	2	9.09

Envisioning future change	Requiring a standardization	9	40.91

#### 4.2.1. Theme 1: Navigating Challenges

This theme was defined as challenges related to teaching and assessing nursing student self‐care competencies, ranging from individual (students and educators) and organizational level challenges, to no challenges at all.

##### 4.2.1.1. Students‐Related Challenges

This category was further grouped into two subcategories, including students’ sociodemographic characteristics and characters/personalities. Students’ diverse sociodemographic characteristics, such as belief system, family background, economic status, cultural background, and educational background were mentioned as student‐related challenges by five participants, exemplified by a comment: “The challenge or obstacle in teaching and assessing self‐care competencies for nursing students is the conditions of students, who come from diverse backgrounds (family, economic, cultural and educational system at the previous level) …” (Respondent 12).

Students’ characters and personalities were mentioned by three respondents as students‐related challenges. Respondent 20 described a generational characteristic that presented challenges in teaching nursing student self‐care in this population group, “The development of students today, who are freer in determining their attitudes and choices,” which may include how they valued self‐care. Respondent 15 added challenges, “…the lack of awareness and sincere intention of students themselves to care for themselves.” Students’ unique personalities were also mentioned by Respondent 3 as another challenge to capture problems encountered by nursing students, especially among those with introverted personalities. These students were naturally more reserved and quieter, which meant it “…isn’t easy to recognize what is being said and what is actually happening.”

##### 4.2.1.2. Educators‐Related Challenges

Five respondents recognized challenges in teaching and assessing nursing student self‐care competencies due to factors associated with the educators. This is particularly related to the specific competencies posed by the nursing educators to teach and assess it to their students: “Teaching and assessment materials vary greatly depending on the knowledge of the lecturer teaching” (Respondent 16), and “Each student has a different capacity for self‐care so creativity among lecturers and supervisors need[s] to be improved to respond to these conditions” (Respondent 12). The respondents also noted the difficulty of teaching particular nursing student self‐care dimensions to students, as expressed by Respondent 2: “Self‐care, especially spiritual and relational, is the most challenging to teach to students.” and “…psychological, emotional, relational and stress care” (Respondent 15).

##### 4.2.1.3. Organizational‐Related Challenges

Organizational‐level challenges emerged as a dominant concept, defined as challenges in teaching and assessing nursing student self‐care competencies due to systemic factors that impact the entire organization. These challenges include inadequate guidelines and excessive/extra workload required to teach the concept. Among these two subcategories, the absence of sufficient guidelines for teaching and assessing nursing student self‐care appeared as a frequently referenced element, illustrated by the statement: “There have not been a clear guideline and road map so we are still having difficulty determining the legal basis that we can use to formally implement it” (Respondent 3).

Four respondents perceived teaching and assessing nursing student self‐care as presenting excessive or extra workload for educators, which served as the second subcategory. “Insufficient time and human resources” were mentioned by Respondent 1, who perceived the currently available number of students and tasks outweighed the educator workforce available. Accordingly, teaching and assessing nurse self‐care for nursing students was seen as additional work that “…demands an extra role of academic and clinical supervisors…” (Respondent 1). This situation further burdened educators who were already overworked, expressed by Respondent 13 as “increasing personnel workload.”

In addition, the organization’s lack of support and commitment also contributed to the educators’ load of teaching and assisting students in fulfilling their self‐care needs. These are evident in the following quote.

The evaluation of student needs often has to conflict with the interests of management/foundations. Support from stakeholder [organization] is also less than optimal so that the ‘burden’ of teaching and meeting student needs is entirely the responsibility of the lecturers (Respondent 18).

##### 4.2.1.4. No Challenges

Four respondents perceived no challenges in teaching and assessing nursing student self‐care competencies. Three respondents expressed this, “In the meantime, there are no obstacles at our place” (Respondent 4), “There is no obstacles…” (Respondent 22) and “None [challenges]” (Respondent 23). One respondent’s comment, however, seemed to suggest that the nonidentified challenges should be viewed as “conditional.” This was because nurse self‐care for students had not been taught formally in their institution, “…so the specific challenges/obstacles cannot yet be identified” (Respondent 5).

#### 4.2.2. Theme 2: Differences in Teaching and Assessment Approaches

The theme highlighted the extent of current teaching and assessment of nursing student self‐care competencies in the participating institutions, ranging from no instruction to informal or formal integration into the curriculum.

##### 4.2.2.1. No Teaching and Assessment

Four participants reported that nursing student self‐care was not currently taught and assessed in their institutions, “…there is no program related to this” (Respondent 11). Others explained, “So far, self‐care material for students has never been provided. The focus of self‐care material is only for clients/patients” (Respondent 1), and “There has not been specific material provided to students and in what semester this course is delivered” (Respondent 14). Likewise, Respondent 21 wrote that the self‐care concept targeting students’ needs in their institution’s curriculum “…has not been implemented well and is not even clear.”

##### 4.2.2.2. Informally Taught and Assessed

Five respondents declared that nursing student self‐care was taught and assessed informally in their institutions, inferring there was no formal or structured learning program. For example, Respondent 19 wrote,

“Currently, the nursing professional study program does not have a curriculum that refers to students’ self‐care, so the nursing study program does not teach this material, because at this stage, students focus on patient practice in the clinical placement sites. Therefore, we teach the topic above if there are related cases in the educational process, such as resilience, then there will be further guidance for the student. Currently there is no structured teaching and assessment for student self‐care.”

Several teaching methods were highlighted by the respondents; for instance, Respondent 5 mentioned “informal in‐class discussions,” and “lecturer–student interactions.” Meanwhile, Respondent 16 identified teaching methods, including “seminar forums,” “reflections,” and “integrated into several institutional courses.” Regardless of the teaching methods, students are expected to be able to internalize and implement the patients’ self‐care concepts/values into their own lives, as illustrated in the following quote: “…students are expected to be able to fulfill client self‐care and are not specifically taught to care for themselves. However, students are expected to be able to integrate client self‐care material into themselves…” (Respondent 5).

##### 4.2.2.3. Taught and Assessed

Nursing student self‐care was claimed to be taught and assessed by two respondents. However, respondents’ responses suggested that the concept was framed in different ways and that the distinction between self‐care for nursing students’ own benefits and self‐care for patient care was not always explicit. Several respondents discussed nursing student self‐care implementation in their institutions primarily in relation to future nursing practice: “It is good and self‐care is very important for students because later it will be applied in the field” (Respondent 24). It is important to teach students self‐care for patients as preparation for service (Respondent 23).

#### 4.2.3. Theme 3: Envisioning Future Change

The final theme that emerged was future directions, which indicated educators’ expectations of how nursing student self‐care should be taught and assessed in the future. Nine respondents (40.91%) suggested the need for “standardization… so that the process is more structured” (Respondent 8), which involves developing strategies and implementing standards‐based methods in teaching and assessing self‐care for nursing students, such as “competencies regarding self‐care… so that they are relevant to the application of self‐care in learning” (Respondent 21), and “a special instrument” (Respondent 9), or “measurable evaluation of all teaching related to student self‐care” (Respondent 15) to evaluate students’ self‐care outcomes continuously.

Respondents also mentioned the important roles of the AINEC and all nursing educators in encouraging students to embrace nursing student self‐care. Following are a few examples of respondents’ comments: “Perhaps, in the future, AINEC can accommodate this because institutional course development is also limited” (Respondent 16) and “Every lecturer has a role in providing material/practice to meet student needs” (Respondent 25).

## 5. Discussion

This study analyzed data generated from an online survey to explore academic coordinators’ perspectives on the current integration of nursing student self‐care in 13 nursing education institutions in Indonesia. The respondents’ responses suggested that nursing student self‐care has been incorporated into the represented institutions, but there are considerable variations and challenges in its implementation. A relatively limited focus on teaching nursing student self‐care appeared to exist. A key feature of the study design that should be considered when interpreting the findings is that the closed‐ and open‐ended questions addressed complementary aspects of the study and were not designed to measure directly comparable information. The open‐ended questions enabled respondents to provide additional comments and raise issues they considered relevant but not have been fully captured by the closed‐ended questions. Instead of comparing the two sets of findings directly, the open‐ended responses were used to enrich and contextualize the interpretation of the closed‐ended question results, and were considered together where applicable throughout the discussion.

The closed‐ended responses revealed that most respondents reported nursing student self‐care was taught to some extent within their institutions, predominantly utilizing formal teaching strategies. These findings aligned with those found in a Canadian‐based study [5], in which teaching nursing student self‐care mostly occurred formally in class. A noteworthy finding from the open‐ended data indicated that respondents relied more heavily on informal than formal teaching methods. Curriculum documents from the institutions represented by the participating academic coordinators were not reviewed, making it difficult to verify exactly which teaching concepts and practices the respondents were referring to. The variations identified between the two findings may be attributed, in part, to the potential for quantitative and qualitative approaches to capture different aspects of a complex phenomenon [38]. Although closed‐ended responses may also be susceptible to social desirability bias [39], variations in respondents’ interpretations of the nursing student self‐care concept may offer another plausible explanation for the discrepancies. Respondents regarded students’ own self‐care as an implicit outcome of patient‐focused self‐care education and as an integral component of professional nurse development, rather than a separate educational domain. This conceptualization may help explain the lack of a clear distinction between formal and informal teaching practices, as well as between patient‐ and student‐focused self‐care content reported by respondents. Similar expectations that students would apply principles of caring for others to care for themselves as a tenet of professional identity formation have been reported in previous studies [40–42]. In addition, although the present findings do not allow definitive conclusions, this apparent discrepancy may reflect the reported absence of explicit standards for teaching nursing student self‐care. Previous publications have described the inconsistent integration of nursing student self‐care within curricula, partly indicating a lack of consensus regarding its conceptualization and targeted competencies [2, 41].

Another notable finding was that despite some of the nursing student self‐care dimensions being rated by the respondents as the most important components to be included in Indonesia’s nursing education curriculum, these aspects were not frequently taught. The nursing student self‐care dimensions perceived as most important were spiritual, psychological, and emotional, comparable to those found in the study by Docherty‐Skippen et al. [5]. The emphasis on spirituality may be considered in light of Indonesia’s strong culture of religiosity and spirituality, where those values have long been central to and shaped Indonesians’ everyday life [43]. The most frequently taught dimensions in the current study were resilience to cope with stressful situations and professional and physical self‐care. In Canada, self‐care dimensions not mandated by regulatory bodies, including relationship, emotional and spiritual, were the least taught [5]. This description may have limited applicability to the context of the present study because nursing student self‐care, in any dimension, has not been standardized through a national framework in Indonesia [20, 44]. A potential explanation is that teaching psychological, emotional, and relationship dimensions were viewed by the respondents as the most challenging dimensions to teach, as evidenced by both the closed‐ and open‐ended data. This lower teaching frequency on these dimensions may also point to the complexity of teaching more abstract or intangible subjects compared with more clearly defined topics [45], such as professional and physical self‐care. The complex nature of the more abstract self‐care dimensions may make them more likely to be integrated across broader educational experiences [46]. As such, respondents may have been less likely to identify them as standalone topics, potentially contributing to lower reported teaching frequency. These findings may also be consistent with a curriculum that has a patient‐centered orientation. In this context, greater emphasis may be placed on the resilience, and professional and physical self‐care dimensions because they were perceived to have more direct and observable implications for students’ capacity to provide quality nursing care. This interpretation aligns with recent scholarly discourse highlighting the urgency of incorporating nursing student self‐care into education, with a stronger rationale for preparing students for their future professional roles [47, 48].

Most respondents expressed satisfaction with how nursing student self‐care was taught and assessed in their institutions, with teaching satisfaction generally scored higher than with assessment satisfaction. Although further research is warranted, this pattern may reflect respondents’ perception that assessing nursing students’ self‐care competencies was more challenging than teaching them. This finding is consistent with previous research among Canadian nursing program directors [5], suggesting broader educational challenges in integrating nursing student self‐care across nursing education contexts. Establishing causal relationships was beyond the scope of this study, yet, possible explanations may be considered from educational and contextual perspectives. Nursing student self‐care is a complex, multifaceted competency that integrates cognitive, affective, and behavioral dimensions, as well as personal growth and professional identity formation [5, 41]. While nursing student self‐care concepts may be woven into routine educational activities, evaluating students’ development of these dimensions may present additional challenges. This is because assessment requires explicit learning outcomes and appropriate evaluation methods that better reflect the complexity of the competencies [5, 49]. This interpretation is supported by literature suggesting that assessing multidimensional professional competencies may not be fully captured through conventional assessment approaches [50, 51]. Given the context of the present study, this finding may also be partly explained by the lack of explicit integration of the concept into the national curriculum framework [20]. Without clearly defined standards, the integration of nursing student self‐care into teaching practices may be shaped by institutional priorities and educators’ professional interpretations and discretion [2, 24, 52]. This flexibility, however, may inadvertently contribute to variation and uncertainty in how the competencies are evaluated [2, 5, 50].

Exploring intention–action gap values may further enrich our understanding of the study’s findings. All dimensions yielded positive results, supporting the teaching of more nursing student self‐care content across all dimensions to students at respondents’ institutions. The capacity to navigate self‐care resources showed the highest intention–action gap, but this score may be more appropriately viewed as inadequate facilitation of students’ practical self‐care skill development rather than a complete absence of its teaching. In particular, it may indicate that, while the represented educational institutions exposed students to nursing self‐care concepts, they offered inadequate support in developing students’ capacities to determine where and how to access, understand, appraise, and utilize appropriate self‐care resources when needs arise [2, 41]. While further investigation is needed, this finding may also reflect individual and organizational challenges in teaching and assessing nursing student self‐care, as perceived by academic coordinators. These identified multilevel barriers, including diverse student characteristics, variations in educators’ skills and knowledge, inadequate guidelines, and heavy workloads, are consistent with the relatively small body of literature on self‐care and well‐being education among nursing and healthcare students from the perspectives of educators and other key educational stakeholders [5, 53].

The study’s findings concerning the educational approach, including the reported predominance of educator‐guided instructional methods and the relatively limited use of experiential learning strategies, may also warrant further discussion. Educator‐guided instructional approaches, such as lectures, may facilitate students’ knowledge acquisition, collaborative learning, and interactions with educators. However, they may be less well suited to foster experiential and reflective learning processes that support the application of theoretical knowledge and behavioral change [54–56]. This may limit opportunities for students to reflect on their own experiences, identify their self‐care needs, explore available resources, develop contextually meaningful self‐care strategies, and translate these into everyday practice [41, 47]. The findings, considered alongside existing evidence, indicate the potential value of strengthening experiential approaches in nursing education. Teaching strategies such as simulation, role‐play, and reflective practices may support students’ development of self‐care competencies by enabling them to actively engage with the concept and transfer it into practice [41]. Given their limited use among the represented institutions, future exploration of how these approaches can be effectively integrated into nursing curricula warrants investigation.

To help interpret the findings within the study’s theoretical framework, it is worth examining the challenges associated with integrating nursing student self‐care competencies. Respondents identified numerous challenges, mirroring the complexity of the phenomenon, which involved dynamic interactions between individuals and their broader contextual factors [1]. Organizational‐level challenges dominated responses, especially about insufficient guidelines for teaching and assessing nursing student self‐care competencies. This together with the limited availability of pro‐nurse self‐care policies and lack of explicit inclusion of nursing student self‐care content in the curriculum may also reflect a broader professional context, in which client‐oriented values have traditionally characterized the Indonesian nursing practice and education [20, 44]. This longstanding emphasis on caring for others has also been highlighted by Dossey [1] and other scholars in nursing literature [2, 57], with recent reviews calling for more explicit integration of nursing self‐care into pre‐licensure nursing education [41, 47, 55].

Using the TIN as a conceptual lens, particularly its collective dimensions [27], the current study’s findings indicate that the observed challenges may reflect a dynamic interplay between structural (the “It” space of TIN) and cultural (the “We” space of TIN) dimensions rather than occurring separately. The findings suggest that structural circumstances may reduce opportunities for the systematic inclusion of nursing student self‐care across education institutions. The findings corroborate earlier studies, demonstrating that structural factors, including educators’ heavy workloads, staffing imbalances, perceived competency gaps, competing institutional priorities, and a lack of clearly articulated curriculum guidance, are pervasive across nursing education systems irrespective of geographical locations [2, 47, 55]. The academic coordinators’ responses could also be interpreted as reflecting shared professional cultures and norms embedded within the educational environment. A prevailing value that prioritizes caring for others, and an implicit expectation that students will cultivate self‐care capacities through learning to care for patients, may influence the extent to which nursing student self‐care is valued and considered legitimate. These findings imply that addressing challenges in embedding nursing student self‐care may benefit from simultaneously considering both structural and cultural factors, and acknowledging that they may intersect. Structural changes such as greater curricular integration, explicit curriculum frameworks, educator development, access to self‐care resources, and institutional commitment, may provide structural support for nursing student self‐care integration within the education context [2, 41, 47]. However, isolated structural reforms may be insufficient to achieve sustained and meaningful integration if the underlying cultural norms that discourage nursing student self‐care remain unchanged [58]. Instead, structural reform and cultural transformation should be aligned and addressed synergistically to create supportive learning environments where nursing student self‐care is not only taught but also valued and reinforced, as it is explicitly embodied in everyday educational and institutional practices [2, 41, 58]. Such environments encourage nursing students to embrace their self‐care as both personal and professional responsibilities and provide opportunities to develop the competencies needed to foster sustainable nursing practice. This interpretation is consistent with TIN, which proposes that meaningful transformation requires attention to multiple dimensions of reality represented within the four quadrants of TIN [1]. Accordingly, meaningful and sustainable integration of nursing student self‐care requires alignment across individual, collective, cultural, and structural dimensions [27, 59].

### 5.1. Strengths and Limitations

This study has some limitations. Considering this was a cross‐sectional study, causal relationships between variables could not be explored. This study also relied on self‐reporting methods to collect data, which may have led to bias. Its limited scope may also limit generalizability. The potential for conceptual ambiguity between nursing student‐ and patient‐focused self‐care is acknowledged. Despite explicit clarifications throughout the participant information sessions and study materials, some respondents appeared to interpret the two concepts interchangeably. The findings represent individual coordinators’ perceptions and should not be interpreted as definitive measures of institutional attributes, practice, or policy. Finally, responses from respondents within the same institution due to shared institutional experiences may not be entirely independent and should be considered when interpreting the findings. Despite these limitations, this study provides valuable insights into current educational approaches to nursing student self‐care within the participating Indonesian institutions. It also addresses an important gap in the literature by examining the perspectives of key academic stakeholders, offering insights into the structural and practical aspects of integrating nursing student self‐care. The use of TIN as the theoretical framework informed the inclusion of both quantitative and qualitative approaches, enabling the collection of complementary data and facilitating a broader understanding of the phenomenon.

## 6. Conclusion

Nursing student self‐care research has largely been viewed through the lenses of students and frontline educators. This study contributes to the literature by examining how nursing education institutions help students develop self‐nurturing behaviors from the perspective of academic coordinators, grounded in integral nursing principles. While this study offers valuable insights into the perspectives of participating key academic stakeholders, the findings should be considered within the scope and limitations of the study. This includes the inclusion of 13 institutions rather than a nationally representative sample and the reliance on cross‐sectional self‐reported data from one stakeholder group.

The findings reveal a need for greater emphasis on teaching nursing student self‐care within the Indonesian nursing institutions represented in this study. Variations in findings also emerged, potentially reflecting the complex interplay between structural and cultural dimensions that shape the integration of nursing student self‐care into education. Nursing education institutions may benefit from explicit curriculum frameworks that articulate learning objectives, content, pedagogical approaches, and assessment strategies for nursing student self‐care, since they are obligated to create an organizational culture that embodies and promotes nursing student self‐care. This involves aligning institutional curricula, policies, and practices with self‐care values, and ensuring the provision of inclusive and accessible resources. Future research should incorporate the perspectives of multiple stakeholders, including students, educators, clinical instructors, and representatives of nursing regulatory bodies or professional nursing associations, to gain a more comprehensive understanding of the phenomenon. Given that self‐care integration is shaped by interconnected structural and cultural factors, future research employing longitudinal and nationwide approaches is warranted. Studies comparing reported practice with current institutional curriculum documents would help determine whether perceptions align with the formal educational program and thus verify the accuracy of self‐reported data. Future research should also evaluate the effectiveness and meaningfulness of different pedagogical and assessment approaches in developing nursing students’ self‐care competencies during preregistration education and into professional practice.

## Author Contributions

Made Rini Damayanti: conceptualization, methodology, formal analysis, investigation, data curation, writing–original draft, writing–review and editing, visualization, project administration, and funding acquisition. Kaori Shimoinaba: conceptualization, methodology, formal analysis, writing–review and editing, and supervision. Katrina Recoche: conceptualization, methodology, formal analysis, writing–review and editing, and supervision.

## Funding

This study was supported by the Indonesian Government’s Endowment Funds for Education (LPDP) Agency (No. 202203223108182).

Open access publishing facilitated by Monash University, as part of the Wiley ‐ Monash University agreement via the Council of Australasian University Librarians.

## Conflicts of Interest

The authors declare no conflicts of interest.

## Data Availability

Data supporting this study’s findings can be obtained from the corresponding author upon reasonable request.
